# Rapid Development of Clinically Symptomatic Radiation Recall Pneumonitis Immediately Following COVID-19 Vaccination

**DOI:** 10.7759/cureus.14303

**Published:** 2021-04-05

**Authors:** Cole R Steber, Janardhana Ponnatapura, Ryan T Hughes, Michael K Farris

**Affiliations:** 1 Radiation Oncology, Wake Forest School of Medicine, Winston-Salem, USA; 2 Radiology, Wake Forest School of Medicine, Winston-Salem, USA

**Keywords:** radiation recall pneumonitis, covid-19, covid-19 vaccine, immunotherapy, non-small cell lung carcinoma (nsclc), oligometastatic, nsclc

## Abstract

In this report, we present the case of a 66-year-old man who received local consolidation radiotherapy to the right lung and mediastinum for oligometastatic non-small cell lung cancer (NSCLC) following partial response to upfront chemoimmunotherapy. He continued with maintenance immunotherapy and was asymptomatic for eight months after completing radiation therapy. He then developed symptoms consistent with pneumonitis within three to five days of his first administration of the coronavirus disease 2019 (COVID-19) vaccine injection. He reported that these symptoms significantly intensified within three to five days of receiving his second dose of the vaccine. The clinical time frame and radiographic evidence raised suspicion for radiation recall pneumonitis (RRP). Patients undergoing maintenance immunotherapy after prior irradiation may be at increased risk of this phenomenon that may be triggered by the administration of the COVID-19 vaccine.

## Introduction

Radiation recall is described as a delayed inflammatory reaction occurring within tissues that have been previously irradiated [1]. This "recall" inflammatory episode is rare but can be classically triggered by chemotherapy agents such as anthracyclines, taxanes, and gemcitabine [2]. The reaction most commonly involves the skin but it has also been reported within other organs including the lungs [3]. 

Radiation recall pneumonitis (RRP) may be distinguished from acute radiation pneumonitis based on the acute development following the administration of a triggering agent [3]. In addition to the chemotherapy agents described above, there have been reports of the development of RRP after the use of targeted agents such as tyrosine kinase inhibitors and immune checkpoint inhibitors (ICI) [1,4]. 

The coronavirus disease 2019 (COVID-19) pandemic has brought about many unique challenges to the care of patients with cancer. There has been a recent case report that discussed COVID-19 triggering a possible acute onset of RRP in a patient irradiated 3.5 years ago [5]. Similarly, radiation recall dermatitis has been reported following the administrations of the Pfizer-BioNTech vaccine for COVID-19 (Pfizer Inc., New York, NY; BioNTech SE, Mainz, Germany) [6]. In this report, we discuss the case of a possible RRP developing rapidly after the administration of the Moderna COVID-19 vaccine (Moderna, Inc, Cambridge, MA). To the best of our knowledge, this report would represent the first description of a radiation recall event related to this vaccine.

## Case presentation

A 66-year-old man was diagnosed with oligometastatic non-small cell lung cancer (NSCLC) in January of 2020. MRI of the brain did not reveal any metastatic disease. Staging with positron emission tomography/CT (PET/CT) demonstrated a large bilobed mass of the right upper lobe (RUL) with the dominant superior mass at 5.4 x 4.2 cm abutting the pleura and a more inferior mass measuring 4.6 x 4.2 cm, as well as multiple involved hilar and mediastinal nodes that measured up to 1.8 cm in short axis. His only site of distant disease was a biopsy-confirmed 1.3-cm rounded lesion in the pancreas. Pathologic subtyping was listed as "not otherwise specified". Molecular analysis revealed no targetable mutations with a programmed death-ligand 1 (PD-L1) expression of 0%. He completed four cycles of first-line systemic therapy with carboplatin, pemetrexed, and pembrolizumab and had no clinically significant side effects due to the therapy. Repeat MRI of the brain and PET/CT demonstrated no brain metastases, with a partial response in the RUL and mediastinum with the dominant RUL mass decreased to 3.4 x 2.7 cm and the inferior mass to 3.3 x 3.2 cm as well as complete response in the pancreatic metastasis with no measurable disease. He was enrolled into an institutional clinical trial (NCT03867175) in which he was randomized to local consolidation radiotherapy to all residual avid disease in the chest followed by the resumption of standard maintenance pemetrexed and pembrolizumab every three weeks.

**Figure 1:**
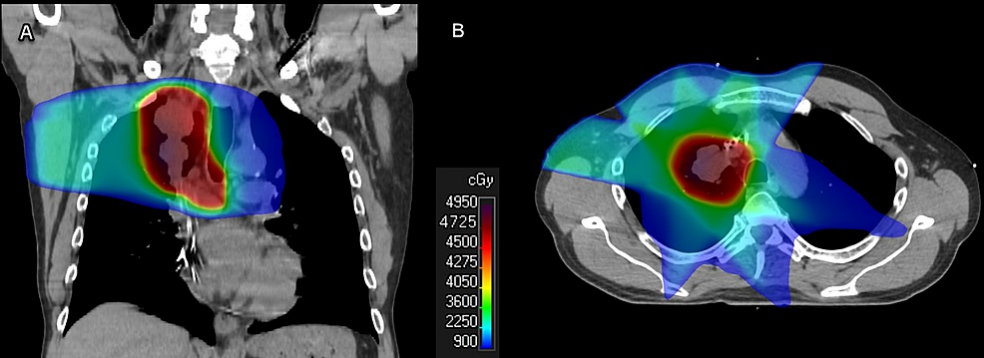
Radiation therapy treatment volumes Coronal (A) and axial (B) representative slices from the original radiation therapy plan delivering 45 Gy in 15 fractions

He completed 45 Gy in 15 fractions in July 2020, as shown in Figure 1, and subsequently resumed pemetrexed and pembrolizumab 15 days after the completion of the radiotherapy. He tolerated the radiotherapy well without any noticeable side effects aside from Common Terminology Criteria for Adverse Events (CTCAE) version 5.0 grade 1 fatigue. Maintenance pemetrexed and pembrolizumab were continued every three weeks for the next eight months with minimal nausea and fatigue. Serial surveillance CT imaging during this time showed the decreasing size of the RUL/mediastinal tumors and the development of grade 1 radiation fibrosis. In November of 2020, there was an interval appearance of a few small scattered multifocal ground-glass opacities with tiny nodular consolidations involving the bilateral lower lobes. These were thought to possibly represent infectious or inflammatory changes and found to be completely resolved on his subsequent CT chest three months later.

**Figure 2:**
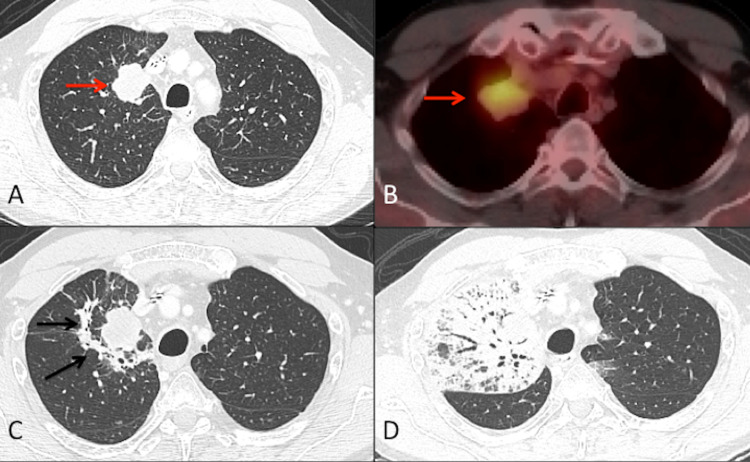
Pre- and post-irradiation imaging Axial section CT chest lung window (A) and axial section fused FDG PET/CT image (B) show lung mass in the upper lobe of the right lung with hypermetabolic uptake of FDG (red arrow) suggesting lung cancer. Axial section of follow-up CT chest (C) after five months of radiation therapy shows early post-radiation fibrosis surrounding the treated mass (black arrows). Follow-up CT chest (D) on March 18, 2021, at eight months after the completion of radiation therapy and after the administration of the second COVID-19 vaccine. The image shows extensive consolidation with surrounding ground-glass opacities and bronchiectasis within the prior radiation therapy portal suggestive of “Radiation Recall” type of pneumonitis (corresponding coronal image of Figure 2D within Figure 3) CT: computed tomography; PET: positron emission tomography; FDG: fluorodeoxyglucose; COVID-19: coronavirus disease 2019

**Figure 3:**
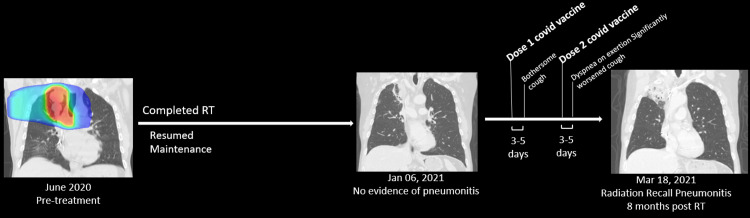
Timeline of events The patient was treated with radiation therapy and then resumed maintenance chemoimmunotherapy. Prior to the administering of the COVID-19 vaccine, there was evolving radiation fibrosis but no evidence of pneumonitis. Clinical symptoms of radiation pneumonitis began shortly after the first administration of the COVID-19 vaccine. CT imaging performed after the second dose of the COVID-19 vaccine revealed significant findings consistent with pneumonitis within the region of the previously irradiated lung COVID-19: coronavirus disease 2019

The patient was tested for COVID-19 as part of pre-procedural protocols, with negative results on June 19, 2020, and March 5, 2021. He received his first dose of the Moderna COVID-19 vaccine (Moderna, Inc, Cambridge, MA) on January 26, 2021, and approximately three to five days after the administration, he developed a bothersome nonproductive cough. He had no other adverse reactions related to the vaccination that he could remember. He explicitly denied arm pain, fevers, chills, weakness, hemoptysis, joint aches, or gastrointestinal (GI) distress. He continued his therapy with pemetrexed and pembrolizumab. At his next cycle on February 23, he reported to his medical oncologist about a persistent bothersome cough. His second dose of the COVID-19 vaccine was delivered on February 25, and he noted that his cough became progressively more severe and frequent around February 28-29. Associated symptoms included increased dyspnea on exertion. These symptoms continued into March 2021. Based on these symptoms, he presented to the clinic for further evaluation, and CT imaging demonstrated interval development of exuberant pneumonitis confined primarily within the prior radiotherapy treatment field (Figure 2). His imaging was interpreted by a board-certified radiologist specializing in thoracic oncology. Given the timeline (Figure 3), now at eight months post-irradiation and following administrations of the COVID-19 vaccine, this was thought to indicate RRP. The patient was started on prednisone 40 mg daily with a long taper, which quickly improved his symptoms. He did not receive any antibiotics as a part of his management. 

## Discussion

The radiation recall phenomenon was first described in 1959 with respect to dermatitis developing after actinomycin D was administered in an area of prior radiation fields [7]. One of the earliest reports of RRP was presented in 1976 by McInerney et al. in a pediatric patient who had been given adriamycin [8]. Due to the rarity of this inflammatory reaction, its precise incidence is not known. In the lungs, it is a clinical diagnosis based on the criteria of acute development of inflammation in previously irradiated parenchyma after a triggering systemic event. Clinical symptoms may include a dry cough, low-grade fever, and shortness of breath [3]. Typical triggers include pharmacological agents, particularly chemotherapies, but nowadays it is increasingly described after the administration of targeted agents and ICI as well [1,2,4,9].

Radiation pneumonitis is a subacute toxicity that occurs in 10-30% of all patients receiving thoracic radiation therapy [10]. It most commonly occurs between one to six months after the treatment, and specifically most symptomatic or CTCAE grade 2+ episodes occur within four months after completing radiotherapy [11]. The management of symptomatic pneumonitis includes steroid treatment over several weeks and involves a long and slow taper with the escalation of management with supplemental oxygen or hospitalization as indicated [12]. Many patients with locally advanced or metastatic NSCLC are now receiving ICI, which has the potential to cause immunotherapy-related pneumonitis in 3-19% of patients [13]. In the era of COVID-19, there has been a concern with regard to overlapping inflammatory responses in patients on ICI therapy and infection with COVID-19, which might potentiate the development of pneumonitis [14].

In our patient, the possibility that the administration of the COVID-19 vaccine triggered an RRP event cannot be ruled out. In terms of the clinical time frame, the development of acute cough/dyspnea at eight months post-irradiation was outside of the normal window for standard radiation pneumonitis. Additionally, CT imaging at five months post-irradiation showed early fibrotic changes but no evidence of radiation pneumonitis. The correlation between the first onset of symptoms just a few days following the first dose of the COVID-19 vaccine followed by significant worsening of symptoms within a few days of the second dose makes a compelling case for RRP triggered by the COVID-19 vaccine. It is also unlikely that this phenomenon was related to immunotherapy-induced pneumonitis, given the CT findings were primarily limited to regions of the previously irradiated lung (as demonstrated in Figure 2).

Though infectious etiologies leading to a radiation recall event are uncommon, there has been a recent report describing the onset of infection with COVID-19 triggering an episode of RRP [5]. This may be explained by a shared underlying mechanism of radiation recall phenomena through an inflammatory reaction to pharmacologic or infectious agents in the cells of irradiated tissues. It is known that infection with COVID-19 can cause immunologic reactions such as cytokine storm or multisystem inflammatory syndrome in children [15,16]. Much like the actual infection, the vaccines engineered to protect against a possible future infection must induce an immune response [17]. The inflammatory state created by the vaccine may promote the development of radiation recall as described in the case report about a dermatitis reaction following the administration of a COVID-19 vaccine in the skin of previously irradiated areas as well as the RRP observed in our patient [6].

The continued vaccination of large numbers of the population may reveal more cases of radiation recall phenomena and allow for the increased scrutiny of the mechanisms, timing, and severity of these episodes. In our particular case, the patient’s pneumonitis was successfully managed with steroids, and we would not discourage any patient who has previously had thoracic irradiation or who is currently on an ICI from obtaining the COVID-19 vaccine at this time.

## Conclusions

We presented the case of a patient who presented with a clinical syndrome consistent with RRP eight months post-radiation therapy for local consolidation of oligometastatic NSCLC, which had developed rapidly after the administration of the COVID-19 vaccine. While it is impossible to establish a direct causal relationship between the COVID-19 vaccine and this patient’s pneumonitis, the timeline of events was atypical of acute radiation pneumonitis. Given the onset and worsening of symptoms within days of the first and second doses of the COVID-19 vaccine, respectively, it is possible that the inflammatory response to vaccination served as a trigger for RRP in this patient.

Oncology providers should be aware of the possibility of the development of RRP in patients who receive the COVID-19 vaccine and have previously received thoracic radiotherapy, particularly those who are also concurrently receiving immunotherapy or chemotherapy. In our view, the benefit of protecting against severe COVID-19 infection in this patient population far outweighs the very small risk of developing this rare event. Hence, the authors strongly caution against deviating from established public health guidelines to vaccinate this high-risk group of patients based on this report alone. Considering that the global vaccination program is currently underway, it is important that other instances of RRP triggered by the COVID-19 vaccine are reported and studied closely.
